# Dental fluorosis and its association with molar incisor hypomineralization in children attending public schools: a cross-sectional study, São João da Boa Vista, 2023

**DOI:** 10.1590/S2237-96222026v35e20251105.en

**Published:** 2026-06-19

**Authors:** Giorgia dos Reis Doval, Sthefany Merlo Valverde Guezin, Letícia Mazeto Previero, Caio Luiz Lins-Candeiro, Gerson Aparecido Foratori-Junior, Luiz Renato Paranhos, Cristiane Maria da Costa Silva

**Affiliations:** 1 Centro Universitário das Faculdades Associadas de Ensino Faculdade de Odontologia São João da Boa Vista SP Brazil Centro Universitário das Faculdades Associadas de Ensino, Faculdade de Odontologia, São João da Boa Vista, SP, Brazil; 2 Universidade de São Paulo Faculdade de Odontologia de Bauru Bauru SP Brazil Universidade de São Paulo, Faculdade de Odontologia de Bauru, Bauru, SP, Brazil; 3 Universidade Federal de Uberlândia Faculdade de Odontologia Uberlândia MG Brazil Universidade Federal de Uberlândia, Faculdade de Odontologia, Uberlândia, MG, Brazil

**Keywords:** Prevalence, Fluorosis, Dental, Health Surveys, Molar Hypomineralization, Cross-Sectional Studies, Prevalencia, Fluorosis Dental, Encuestas Epidemiológicos, Hipomineralización Molar, Estudios Transversales

## Abstract

**Objective:**

To assess prevalence and severity of dental fluorosis in schoolchildren and its association with molar-incisor hypomineralization, caries and socioeconomic factors.

**Methods:**

A cross-sectional study was conducted with a probabilistic sample of children aged 7 to 11 years enrolled at public schools in São João da Boa Vista, São Paulo. Three dimensions were considered: environmental exposure; individual and family characteristics; and clinical outcomes. Fluorosis prevalence and severity were assessed using Dean’s index, while hypomineralization was assessed using European Academy of Paediatric Dentistry criteria. Caries was assessed using the decayed, missing or filled teeth index. Two calibrated examiners performed the examinations. Socioeconomic information was obtained from the children’s guardians. Data were analyzed using descriptive statistics and simple and multiple logistic regression, with a 5% significance level.

**Results:**

473 children participated in this study. Fluorosis prevalence was 28.5%. Very mild (11.0%) and mild (7.4%) to moderate (3.4%) and severe (0.8%) forms predominated. Hypomineralization prevalence was 12.7%, and 3.8% of the children presented both enamel defects. Fluorosis was more prevalent in schoolchildren over 9 years old (odds ratio, OR 2.22; 95% confidence interval, 95%CI 1.34; 3.66); in children without molar-incisor hypomineralization (OR 3.22; 95%CI 1.20; 8.64); and among those whose mothers had studied up to high school level (OR 2.15; 95%CI 1.09; 4.23). There was no association with sex, caries experience or other socioeconomic factors.

**Conclusion:**

Fluorosis occurred predominantly in mild forms, associated with the children’s characteristics and maternal education level.

## Resumen

**Objetivo:**

Evaluar la prevalencia y gravedad de la fluorosis dental en escolares y su asociación con la hipomineralización incisivo-molar, la caries y los factores socioeconómicos.

**Métodos:**

Se realizó un estudio transversal con una muestra probabilística de niños de 7 a 11 años matriculados en escuelas públicas de São João da Boa Vista, São Paulo. Se consideraron tres dimensiones: exposición ambiental; características individuales y familiares; y resultados clínicos. La prevalencia y gravedad de la fluorosis se evaluaron mediante el índice de Dean, y la hipomineralización mediante los criterios de la Academia Europea de Odontología Pediátrica. La caries se evaluó mediante el índice de dientes cariados, perdidos y obturados. Dos examinadores calibrados realizaron los exámenes. La información socioeconómica se obtuvo de los tutores. Los datos se analizaron mediante estadística descriptiva y regresión logística simple y múltiple, con un nivel de significancia del 5%.

**Resultados:**

473 niños participaron en este estudio. La prevalencia de fluorosis fue del 28,5%. Predominaron las formas muy leves (11,0%) y leves (7,4%) a moderadas (3,4%) y graves (0,8%). La prevalencia de hipomineralización fue del 12,7%, y el 3,8% de los niños presentaron ambos defectos del esmalte. La fluorosis fue más prevalente en escolares mayores de 9 años (odds ratio, OR 2,22; intervalo de confianza del 95%, IC95% 1,34; 3,66); en niños sin hipomineralización molar-incisiva (OR 3,22; IC95% 1,20; 8,64); y entre aquellos cuyas madres estudiaron hasta la secundaria (OR 2,15; IC95% 1,09; 4,23). No hubo asociación con el sexo, la experiencia de caries u otros factores socioeconómicos.

**Conclusión:**

La fluorosis se presentó predominantemente en formas leves, asociadas a las características del niño y al nivel educativo materno.

**Ethical aspects**

This research respected ethical principles, having obtained the following approval data:

Research ethics committee Centro Universitário das Faculdades Associadas de Ensino

Opinion number 6,140,628

Approval date 25/6/2023

Certificate of submission for ethical appraisal 70686723.3.0000.5382

Informed consent form Obtained from all participants prior to data collection.

## Introduction 

Dental fluorosis is one of the main adverse effects resulting from chronic fluoride ingestion during the period of dental enamel formation [1]. This condition poses a public health challenge in balancing the benefits of fluoride use in controlling dental caries with the need to minimize risks associated with excessive exposure [2,3]. This dilemma is particularly relevant in preschool children, who are more susceptible to dental fluorosis because they are in the enamel mineralization phase and have a higher risk of inadvertent ingestion of fluoridated products, such as toothpaste [4-6].

Fluorosis severity depends on both the amount and duration of fluoride exposure, and can manifest itself as anything from minor changes in enamel translucency to structural defects with significant aesthetic and functional impact [1,7-9]. Beyond clinical manifestations, this condition can compromise perception of oral health and negatively affect the self-esteem of children and adolescents, representing a challenge that goes beyond the biological sphere and involves the subjective dimensions of well-being [9,10].

Despite the risk of fluorosis, fluoride continues to be fundamental in preventing caries, as it acts in the balance between enamel demineralization and remineralization [6]. This effectiveness justifies its widespread use in different vehicles, such as toothpaste, mouthwash, gel and varnish, and in the fluoridation of public water supplies [6,8]. The challenge, therefore, does not lie in a single source, but rather in controlling cumulative exposure to fluoride from various vehicles, including oral hygiene products and water fluoridation [11].

Epidemiological surveillance is fundamental; by monitoring dental fluorosis prevalence and severity, information can be obtained on the level of population exposure to fluoride. Thus, it is possible to identify situations of excessive ingestion and guide adjustments in fluoridation policies and other preventive strategies, in order to ensure a balance between benefits and risks [11,12].

In Brazil, in 2010, dental fluorosis prevalence was recorded at 17%, reaching 19% in the Southeast region of the country [13]. This data is particularly relevant because this region concentrates greater number of municipalities with fluoridated water supply and, at the same time, better oral health indicators in relation to caries [14]. In addition, the high prevalence of molar incisor hypomineralization, a qualitative enamel defect of multifactorial origin, is noteworthy. Like fluorosis, it manifests itself clinically through alterations in translucency and structural losses, making teeth more susceptible to demineralization and accelerated caries development [15].

These two conditions can coexist, although they have distinct etiologies, which can generate biases in epidemiological estimates [15,16]. The occurrence of clinical overlap between enamel defects in Brazilian populations [17,18] reinforces the need for precise diagnostic criteria to avoid misclassifications in epidemiological surveys.

The experience of dental caries reflects patterns of fluoride exposure, which influence the balance between demineralization and remineralization of dental enamel [6]. Similarly, sociodemographic characteristics, such as age, sex, family income and parental education, can shape health behaviors, including use of and access to fluoridated products, which contributes to variations in such exposure [19,20]. However, dental fluorosis was not monitored in the most recent national survey, conducted in 2023 [21]. This fact reinforces the importance of regional studies to understand the current panorama of the condition and to support public policies that ensure both control of dental caries and prevention of dental fluorosis.

The objective of this study was to evaluate dental fluorosis prevalence and severity in public school students in São João da Boa Vista, São Paulo, as well as its association with molar incisor hypomineralization, caries and socioeconomic factors.

## Methods 

### Design

This is a cross-sectional study developed in accordance with World Health Organization (WHO)recommendations [22]. The study was structured based on the organization of variables into three dimensions [23]: environmental exposure to fluoride, represented by the type of household water supply; children and family member characteristics, including family income and parental education; and clinical outcomes, composed of molar incisor hypomineralization, caries experience and fluorosis, the latter being considered the outcome variable.

### Setting

The research was conducted in public schools in the urban area of ​​São João da Boa Vista, São Paulo state, between October and December 2023. The municipality is located approximately 220 km from the state capital, São Paulo city. In 2022, its population was 92,547 inhabitants, its municipal human development index was 0.79 and its Gini index was 0.51. Regarding infrastructure, 92.2% of households had adequate basic sanitation [24], and the public water supply was universal and fluoridated. 

### Participants

The sample consisted of students attending elementary public schools in the urban area of ​​São João da Boa Vista, São Paulo, aged between 7 and 11 years.

The request for authorization to conduct the examinations with the children was sent to their parents or guardians. Students whose parents or guardians authorized their participation were included in the sample, provided all their four first permanent molars had erupted, a characteristic observed at the time of the epidemiological examination. Molars were considered erupted when the occlusal surface was exposed, without gingival coverage.

The study excluded children with syndromes or conditions affecting tooth formation (e.g., Down syndrome, ectodermal dysplasia and amelogenesis imperfecta); those with extensive restorations or severe wear that prevented assessment; those with intrinsic tetracycline stains or extensive non-removable extrinsic stains; those using fixed orthodontic appliances; or those absent on the day of the examination.

### Sample size and selection 

The sample size calculation was based on a 95% confidence level, estimated 50% fluorosis prevalence, 5% error, and a design effect of 1.2 due to cluster sampling. The estimated minimum sample size was 415 students, plus 20% to compensate for refusals, totaling 498 expected participants.

The selection of schools was carried out by random draw. The municipality was divided into five administrative regions (excluding the industrial district), with all schools identified and drawn proportionally to the number of students enrolled in each area. The central region, being the most populous, was represented by more than one institution. In the end, six schools were selected, ensuring representation of the municipality’s urban area.

### Variables

The primary outcome variables were the presence and severity of dental fluorosis (Dean’s index) [19]. Teeth were classified into six categories, according to Dean’s index [22], namely:

- normal (0) – absence of alteration;
- questionable (1) – enamel shows a slight difference compared to normal enamel translucency, which can vary from a few white flecks to occasional white spots;
- very mild (2) – small opaque and whitish areas, involving less than 25% of the tooth surface;
- mild (3) – more extensive opaque areas, but affecting less than 50% of the surface;
- moderate (4) – enamel with diffuse stains and wear, may include brownish pigmentation; and
- severe (5) – marked hypomineralization, with changes in tooth shape, extensive brown staining, and sometimes structural defects as well.

Dental fluorosis was defined as the presence of any degree of enamel alteration, whereby individuals with a Dean’s index score >0 were considered to be cases [25]. The inclusion of milder degrees of fluorosis was methodologically consistent with previous studies that investigated association between dental fluorosis and molar incisor hypomineralization [16,26] and other enamel defects [27]. This contributed to a more accurate assessment of the interrelationships between enamel defects.

The independent clinical variables were caries experience according to WHO methods using the decayed, missing or filled teeth index [22], and molar incisor hypomineralization, assessed according to European Academy of Pediatric Dentistry criteria [28]. Cases of molar incisor hypomineralization were considered to be when at least one permanent first molar showed well-defined opacities (white, yellow or brownish) larger than 1 mm; post-eruptive fracture; atypical restorations; and extraction attributed to molar incisor hypomineralization. These alterations needed to be present in at least one permanent first molar, and might or might not involve incisors.

The sociodemographic variables were sex, age, family income, parental education level and access to treated water.

### Data sources and collection procedures 

The clinical examinations were conducted in the schools following WHO operational recommendations for epidemiological surveys [22]. The examinations were performed under natural lighting, with the child and examiner seated. A No. 5 plane mirror, wooden spatulas and a periodontal probe with a spherical tip (WHO probe) were used, the latter only to remove visible debris. Prior to examination, the children underwent supervised brushing. All teeth present in the oral cavity were assessed, both deciduous and permanent. However, molar incisor hypomineralization classification was performed exclusively on permanent teeth. Two trained and calibrated examiners conducted the examinations. The data were recorded on specific forms.

The sociodemographic variables were obtained through a structured questionnaire, sent to and answered by parents or guardians. The instrument included basic information about the children (sex and age) and family characterization data, such as monthly family income, categorized according to minimum wage ranges, parental education level, classified into formal education levels, and type of household water supply (water mains/treated water, well/spring, other).

### Efforts to minimize bias

In order to reduce diagnostic bias, teeth were classified as normal in cases of doubt regarding the presence of fluorosis. The same procedure was adopted for caries and molar incisor hypomineralization, in order to avoid overestimating prevalence. The examiners underwent a training and calibration process, consisting of theoretical and practical stages.

Initially, a theoretical stage was conducted, involving a preliminary study of the indices used, analysis of clinical images projected on slides, and discussion of situations that could be encountered during fieldwork. This stage aimed to standardize the diagnostic criteria for fluorosis, molar incisor hypomineralization and dental caries between the examiners. Subsequently, practical training took place using a standardized intraoral image bank representing different clinical conditions of fluorosis, caries and hypomineralization.

During training, the examiners recorded their diagnoses on the same standardized clinical forms used in the study itself, and any discrepancies identified were discussed until consensus was reached. The training totaled eight hours. After this stage, clinical calibration was performed. For this, the examiners evaluated 25 children with mixed dentition, selected by convenience from a school not randomly chosen to compose the final sample.

After a one-week interval, the same children were reassessed to estimate inter- and intra-examiner agreement. Diagnostic reproducibility was measured using the Kappa coefficient, which indicated high inter-examiner agreement for caries (Kappa 0.81), fluorosis (Kappa 0.89) and molar incisor hypomineralization (Kappa 0.91). Intra-examiner Kappa values ​​were greater than 0.81.

### Data analysis

Descriptive analyses were performed using absolute and relative frequencies. Following this, simple and multiple logistic regression models were fitted to analyze associations with the dental fluorosis outcome. The model quality was analyzed using the Akaike Information Criterion. Unadjusted and adjusted odds ratios (OR) were then estimated, with their respective confidence intervals (95%CI). All analyses were performed using the R program (R Core Team, 2025).

## Results

Six public schools in the urban area of ​​São João da Boa Vista, São Paulo, were randomly selected, ensuring representation of the different administrative regions of the municipality, with the exception of the industrial district. A total of 738 children aged 7 to 11 years were enrolled at these institutions and were therefore eligible for the study. Of these, 498 were randomly invited to participate through a simple draw. The number of participants per school was proportional to the total number of enrolled students. The final sample consisted of 473 schoolchildren, all with complete data for fluorosis, molar incisor hypomineralization and caries (Figure 1). This resulted in a response rate of 94.9%.

**Figure 1:**
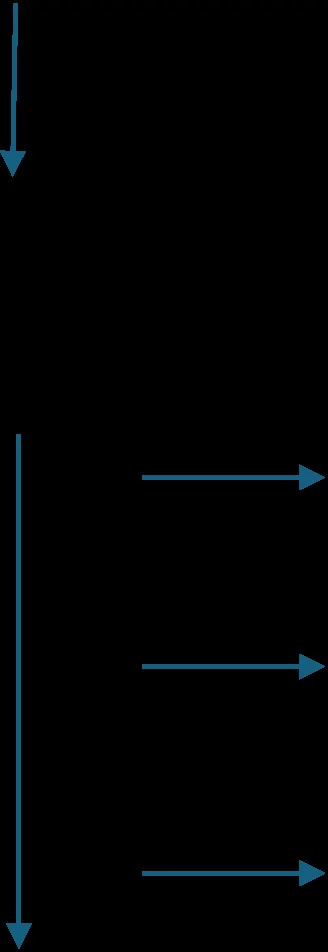
Study sample selection. São João da Boa Vista, 2023 (n=473)

The average age of the participants was 8.9 ± 1.2 years, ranging from 7 to 11 years (median 9 years), with 52.6% being male. Most families had income of between 1 and 3 minimum wages (53.5%), and a large proportion of mothers had completed high school (48.7%). Regarding household water supply, 86.0% reported using mains water treated by the São Paulo State Basic Sanitation Company, while 6.8% consumed well or spring water (Table 1).

**Table 1:** Descriptive analysis of the sociodemographic profile variables of the sample expressed in absolute (n) and relative (%) frequencies. São João da Boa Vista, 2023 (n=473)

Variables	Frequency n (%)
**Sex **	
Female	224 (47.4)
Male	249 (52.6)
**Age **(years)	
7	68 (14.4)
8	120 (25.4)
9	110 (23.3)
10	111 (23.5)
11	64 (13.5)
**Monthly family income **(**minimum wages**)	
No income	21 (4.4)
Up to 1	113 (23.9)
1-2	156 (33.0)
More than 2-3	97 (20.5)
More than 3-5	39 (8.2)
More than 5-10	8 (1.7)
More than 10-20	1 (0.2)
More than 20	1 (0.2)
No information	37 (7.8)
**Paternal schooling**	
Illiterate	5 (1.1)
Literate	32 (6.8)
Up to complete 5th grade	26 (5.5)
Up to complete 9th grade	47 (9.9)
Up to complete high school	86 (18.2)
Incomplete higher education	142 (30.0)
Complete higher education	20 (4.2)
No information	115 (24.3)
**Maternal schooling **	
Illiterate	4 (0.8)
Literate	4 (0.8)
Up to complete 5th grade	17 (3.6)
Up to complete 9th grade	82 (17.4)
Up to complete high school	230 (48.7)
Incomplete higher education	45 (9.5)
Complete higher education	54 (11.4)
No information	37 (7.8)
**Water used at home **	
Mains water (treated water)	407 (86.0)
Well or spring	32 (6.8)
Other	3 (0.6)
No information	31 (6.6)

Dental fluorosis prevalence was 28.5%, with very mild forms predominating (11.0%). Moderate (3.4%) and severe (0.8%) cases were also observed. Molar incisor hypomineralization prevalence was 12.7%, and 3.8% of the total sample (13.3% among fluorosis cases) presented both conditions simultaneously (Table 2). Considering both deciduous and permanent dentition, 50.5% of the children had caries experience (Table 3).

**Table 2:** Descriptive analysis of clinical variables: severity of dental fluorosis, molar incisor hypomineralization and caries experience in deciduous and permanent dentition, expressed as absolute (n) and relative (%) frequencies. São João da Boa Vista, 2023 (n=473)

Variables	Frequency n (%)
**Fluorosis severity**	
Normal	338 (71.5)
Questionable	35 (7.4)
Very mild	52 (11.0)
Mild	28 (5.9)
Moderate	16 (3.4)
Severe	4 (0.8)
**Molar incisor hypomineralization **(MIH)	
MIH=0	413 (87.3)
MIH>0	60 (12.7)
**Caries experience in primary dentition **	
dmft^1^=0	277 (58.6)
dmft>0	197 (41.4)
**Caries experience in permanent dentition**	
DMFT=0	389 (82.2)
DMFT>0	84 (17.8)

^1^dmft: primary decayed, missing or filled teeth; ^2^DMFT: permanent decayed, missing or filled teeth.

In the multiple logistic regression analysis, fluorosis was significantly more frequent in schoolchildren:

- older than 9 years old (OR 2.22; 95%CI 1.34; 3.66);
- without molar incisor hypomineralization (OR 3.22; 95%CI 1.20; 8.64); and
- whose mothers had complete high school education (OR 2.15; 95%CI 1.09; 4.23).

Although fluorosis prevalence was higher among children who consumed mains water (29.5%) compared to those who used well or spring water (18.8%), the difference was not significant (p-value 0.201). After adjustment there was no significant association with sex, water source used or caries experience (Table 3).

## Discussion

This study found 28% dental fluorosis prevalence, with a predominance of mild forms. It was observed that the variables in the “individual characteristics” and “clinical outcomes” dimensions showed significant association with the condition. Children over 9 years of age had higher odds of fluorosis, which is explained by the greater number of erupted permanent teeth and, therefore, greater clinical visibility of enamel defects. In the clinical outcomes dimension, inverse association was identified between fluorosis and molar incisor hypomineralization. No associations were observed with sex, caries experience, family income or type of water supply.

This study has certain limitations that should be considered when interpreting the results. Its cross-sectional design does not allow causal relationships to be established, which restricts conclusions regarding the associations observed. Using questionnaires completed by parents or guardians may have introduced information biases resulting from the increased awareness generated by the study itself. Clinical examinations, conducted in a school setting, are also subject to limitations related to lighting and tooth drying, which can influence diagnostic accuracy. The sample only included students from the urban public school system, which reduces the possibility of generalization to other social and geographical contexts.

The methodological rigor adopted, including the calibration process with a high Kappa value, standardized diagnostic criteria and probabilistic sampling, contributes to the solidity of the results found. This study highlighted the need for continuous monitoring of fluorosis, especially in its moderate and severe forms, given its potential aesthetic and psychosocial impact [8,9].

In this study, 4% of children with fluorosis simultaneously presented molar incisor hypomineralization. From an etiological point of view, these are distinct conditions: fluorosis results from excessive fluoride exposure during enamel formation, while molar incisor hypomineralization results from the interaction between systemic factors and genetic predisposition that can interfere with ameloblastic activity during the dental maturation phase [15]. Although both are qualitative enamel defects, they present distinct clinical manifestations. Fluorosis manifests itself as diffuse opacities, symmetrically distributed throughout the dentition, while molar incisor hypomineralization is characterized by well-defined lesions in the enamel, with an asymmetrical distribution. 

The clinical manifestation of both conditions depends on their severity and can range from white opacities in the enamel to structural losses in more severe cases [7,15,28], which makes differential diagnosis challenging when both defects are present. This is a particularly relevant concern in field studies, where the overlap of defects can lead to overestimation or underestimation of prevalence [15-18]. In Mexico, in 2023, this concern was evidenced by demonstrating the inverse relationship between fluorosis and molar incisor hypomineralization in schoolchildren exposed to high concentrations of fluoride in water. Distinct etiological mechanisms are suggested, but which may overlap clinically [16].

In this study, the high Kappa index (≥0.81) demonstrated excellent diagnostic reliability, emphasizing the robustness of the clinical assessments performed. However, lack of rigorous criteria can compromise both epidemiological surveys and definition of preventive and therapeutic strategies [15]. Thus, the coexistence of the two conditions observed in this study highlighted the need for standardized diagnostic protocols and adequate training of examiners in order to ensure reliable estimates and support decisions in collective oral health.

In this analysis, the “household water source used” variable did not show statistically significant association with the occurrence of fluorosis (p-value 0.413). However, in Brazil, in 2019, the water source was identified as an important determinant, especially in areas supplied by artesian wells. In this case, fluorosis prevalence reached 52% [7]. In 2023, in countries in Africa and Asia, it exceeded 90% [29]. On the other hand, even in urban contexts with wide treated water coverage, concentrations close to the ideal are associated with higher prevalence of the condition, with 40% of the population expected to present some degree of fluorosis [30,31]. The homogeneity of the sample in this analysis, with 86% consuming fluoridated water, probably explains why association was not found, although it does not exclude the importance of continuous monitoring of water quality [14,19].

The predominance of mild cases found is consistent with the pattern described for populations exposed to fluoridated water within recommended levels [30]. However, the occurrence of moderate and severe forms draws attention to possible weaknesses in the operational control of fluoridation and to the role of cumulative exposures during childhood [1,32] widely discussed in the literature, but still poorly monitored in Brazil.

The relationship between fluorosis and socioeconomic conditions is multifaceted and context-dependent. In Mexico, in 2014, high fluorosis prevalence was recorded, even in areas with fluoride concentrations in the water within the level considered optimal. This emphasizes that other sources of exposure also contribute to development of dental fluorosis, such as early use of fluoridated toothpaste and consumption of industrialized beverages [32], these being practices that are more common in populations with better socioeconomic conditions. Conversely, water supply fluoridation instability and dependence on groundwater are associated with contexts of lower socioeconomic development, reflecting underlying inequalities that amplify the burden of the condition [19,20,29].

In 2017, also in Mexico, it was identified that occurrence of severe forms of fluorosis in more vulnerable regions was more frequent in children whose mothers had lower education levels (OR 1.71; p-value 0.007) [32], which may be a result of uncontrolled exposures and limited mitigation capacity [33]. In this heterogeneous scenario, maternal education emerges as a relevant social marker of fluoride exposure. Mothers with lower education levels may have less access to information and resources that allow for the proper management of childhood fluoride exposure. 

Even when the concentration of fluoride in drinking water is similar, behavioral differences associated with maternal education can result in distinct patterns of cumulative fluoride exposure throughout childhood [33]. This result challenges the notion that universal policies, such as drinking water fluoridation, are sufficient to guarantee equitable protection highlighting the need for complementary strategies aimed at more socially vulnerable groups are necessary, even in contexts of broad fluoridated water coverage.

In 2010, dental fluorosis prevalence in Brazilian children aged 12 as a whole was 17%, with very mild (11%) and mild (4%) forms predominating [14]. Moderate fluorosis represented less than 2% of the national sample, while the severe form showed residual occurrence [13]. In the Southeast region of Brazil, in 2010, fluorosis prevalence was higher (19.1%). In this study, conducted in São João da Boa Vista in 2023, 28% prevalence was identified, with a more significant proportion of moderate and severe cases than that described for both the country as a whole and the region in 2010. These findings suggest that the environmental dimension and the lack of systematic monitoring of fluorosis may contribute to changes in the epidemiological profile of this condition over time. The fact that there was no investigation of fluorosis in the last national epidemiological survey, conducted in 2023, [21] makes comparisons difficult and limits the understanding of the temporal trend of this condition in Brazil.

The results of this study not only set a baseline for the municipality, but also raise the hypothesis that the fluorosis panorama in Brazil has changed over the last few years, which directly impacts public health. Future multicenter studies and resumption of monitoring as part of national surveys are fundamental for confirming this trend and guiding public health surveillance policies.

The dental fluorosis prevalence found in this study among public school students, predominantly in mild forms, was higher than the national and regional averages, demonstrating a greater proportion of moderate and severe cases. Significant inverse association was identified between fluorosis and molar incisor hypomineralization. This shows that, despite having distinct etiologies, the two conditions can occur simultaneously.

The relationship between dental fluorosis and lower maternal education levels highlights the role of social determinants in the distribution of this condition. These findings emphasize the importance of epidemiological surveillance of dental fluorosis, especially in contexts with widespread fluoridated water coverage, and point to the need for preventive strategies that consider the role of social inequalities and cumulative fluoride exposure during childhood.

## Data Availability

The data used in this research are available at: https://data.mendeley.com/datasets/23dvmb8yt4/1.

## References

[1] Niazi FC, Pepper T. Dental Fluorosis. In: StatPearls [Internet]. Treasure Island (FL): StatPearls Publishing; 2024 [cited 2025 Oct 1st]. Available from: https://www.ncbi.nlm.nih.gov/books/NBK585039/?report=reader

[2] U.S. Public Health Service. Public health service recommendation for fluoride concentration in drinking water for the prevention of dental caries. Public Health Rep. 2015;130(4):318-31. 10.1177/003335491513000408 PMC4547570 26346489

[3] World Health Organization. Inadequate or excess fluoride: a major public health concern [Internet]. Geneva: WHO; 2019 [cited 2025 Oct 1st]. Available from: https://iris.who.int/bitstream/handle/10665/329484/WHO-CED-PHE-EPE-19.4.5-eng.pdf?sequence=1

[4] Wright JT, Hanson N, Ristic H, Whall CW, Estrich CG, Zentz RR. Fluoride toothpaste efficacy and safety in children younger than 6 years: a systematic review. J Am Dent Assoc. 2014;145(2):182-9. 10.14219/jada.2013.37 24487610

[5] Petrović B, Kojić S, Milić L, Luzio A, Perić T, Marković E, et al. Toothpaste ingestion─evaluating the problem and ensuring safety: systematic review and meta-analysis. Front Public Health. 2023;11:1279915. 10.3389/fpubh.2023.1279915 PMC10622757 37927882

[6] Brasil. Ministério da Saúde. Secretaria de Atenção à Saúde. Departamento de Atenção Básica. Guia de recomendações para o uso de fluoretos no Brasil [Internet]. Brasília: Ministério da Saúde; 2009 [cited 2025 Oct 1st]. Available from: https://www.gov.br/saude/pt-br/composicao/saps/brasil-sorridente/publicacoes/guia-de-recomendacoes-para-o-uso-de-fluoretos-no-brasil.pdf/view

[7] Lima IFP, Nóbrega DF, Cericato GO, Ziegelmann PK, Paranhos LR. Prevalência de fluorose dental em regiões abastecidas com água sem suplementação de flúor no território brasileiro: uma revisão sistemática e metanálise. Cien Saude Colet. 2019;24(8):2909-22. 10.1590/1413-81232018248.19172017 31389538

[8] Wong MCM, Zhang R, Luo BW, Glenny AM, Worthington HV, Lo ECM. Topical fluoride as a cause of dental fluorosis in children. Cochrane Database Syst Rev. 2024;6(6):CD007693. 10.1002/14651858.CD007693.pub3 PMC11187792 38899538

[9] Shyam R, Chaluvaiah MB, Kumar A, Pahwa M, Rani G, Phogat R. Impact of dental fluorosis on the oral health related quality of life among 11- to 14-year-old school children in endemic fluoride areas of Haryana (India). Int Dent J. 2020;70(5):340-6. 10.1111/idj.12567 PMC9379179 32358889

[10] Thilakarathne BKG, Ekanayake L, Schensul JJ, Reisine S. Impact of dental fluorosis on the oral health related quality of life of adolescents in an endemic area. J Oral Biol Craniofac Res. 2023;13(3):448-52. 10.1016/j.jobcr.2023.03.015 PMC10199199 37215392

[11] EFSA Scientific Committee, Bennekou SH, Allende A, Bearth A, Casacuberta J, Castle L, et al. Updated consumer risk assessment of fluoride in food and drinking water including the contribution from other sources of oral exposure. EFSA J. 2025;23(7):e9478. 10.2903/j.efsa.2025.9478 PMC12280829 40698337

[12] Taher MK, Momoli F, Go J, Hagiwara S, Ramoju S, Hu X, et al. Systematic review of epidemiological and toxicological evidence on health effects of fluoride in drinking water. Crit Rev Toxicol. 2024;54(1):2-34. 10.1080/10408444.2023.2295338 38318766

[13] Brasil. Ministério da Saúde. Secretaria de Atenção à Saúde. Departamento de Atenção Básica. Coordenação Nacional de Saúde Bucal. Projeto SB Brasil 2010: condições de saúde bucal da população brasileira 2010: resultados principais [Internet]. Brasília: Ministério da Saúde; 2012 [cited 2025 Oct 5]. Available from: https://bvsms.saude.gov.br/bvs/publicacoes/pesquisa_nacional_saude_bucal.pdf

[14] Roncalli AG, Noro LRA, Cury JA, Zilbovicius C, Pinheiro HHC, Ely HC, et al. Water fluoridation in Brazil: regional distribution and accuracy of information on surveillance in municipalities with more than 50,000 inhabitants. Cad Saude Publica. 2019;35(6):e00250118. 10.1590/0102-311X00250118 31291432

[15] Martignon S, Bartlett D, Manton DJ, Martinez-Mier EA, Splieth C, Avila V. Epidemiology of erosive tooth wear, dental fluorosis and molar incisor hypomineralization in the American continent. Caries Res. 2021;55(1):1-11. 10.1159/000512483 33440378

[16] Varela AFM, Pérez AG, Gutiérrez TV, Navarrete KAM, Nieto Sánchez MP. An inverse relationship between dental fluorosis and molar incisor hypomineralization in Mexican schoolchildren in an area with a high concentration of fluoride in drinking water: a cross-sectional study. PLoS One. 2024;19(9):e0310420. 10.1371/journal.pone.0310420 PMC11404798 39283869

[17] Duarte MBS, Carvalho VR, Hilgert LA, Ribeiro APD, Leal SC, Takeshita EM. Is there an association between dental caries, fluorosis, and molar-incisor hypomineralization? J Appl Oral Sci. 2021;29:e20200890. 10.1590/1678-7757-2020-0890 PMC8289254 34287466

[18] Fernandes IC, Forte FDS, Sampaio FC. Molar-incisor hypomineralization (MIH), dental fluorosis, and caries in rural areas with different fluoride levels in the drinking water. Int J Paediatr Dent. 2021;31(4):475-82. 10.1111/ipd.12728 32965763

[19] Rosário BSM, Rosário HD, Vieira WA, Cericato GO, Nóbrega DF, Blumenberg C, et al. External control of fluoridation in the public water supplies of Brazilian cities as a strategy against caries: a systematic review and meta-analysis. BMC Oral Health. 2021;21(1):410. 10.1186/s12903-021-01754-2 PMC8377988 34412623

[20] Ghafari M, Bahadivand-Chegini S, Nadi T, Doosti-Irani A. The global prevalence of dental healthcare needs and unmet dental needs among adolescents: a systematic review and meta-analysis. Epidemiol Health. 2019;41:e2019046. 10.4178/epih.e2019046 PMC6883027 31778605

[21] Brasil. Ministério da Saúde. Secretaria de Atenção Primária à Saúde. Departamento de Estratégias e Políticas de Saúde Comunitária. SB Brasil 2023: Pesquisa Nacional de Saúde Bucal: relatório final [Internet]. Brasília: Ministério da Saúde; 2024 [cited 2025 Oct 5]. Available from: https://bvsms.saude.gov.br/bvs/publicacoes/sb_brasil_2023_relatorio_final_1edrev.pdf

[22] World Health Organization. Oral health surveys: basic methods. 5th ed. [Internet]. Geneva: WHO; 2013 [cited 2025 Oct 01]. Available from: https://iris.who.int/server/api/core/bitstreams/01882193-23ce-4aec-90b5-8647ff99323e/content

[23] Fisher-Owens SA, Gansky SA, Platt LJ, Weintraub JA, Soobader MJ, Bramlett MD, et al. Influences on children’s oral health: a conceptual model. Pediatrics. 2007;120(3):e510-20. 10.1542/peds.2006-3084 17766495

[24] Instituto Brasileiro de Geografia e Estatística. São João da Boa Vista: panorama municipal [Internet]. Rio de Janeiro: IBGE; [cited 2025 Dec 7]. Available from: https://cidades.ibge.gov.br/brasil/sp/sao-joao-da-boa-vista/panorama

[25] Mohd Nor NA, Chadwick BL, Farnell DJJ, Chestnutt IG. The impact of a reduction in fluoride concentration in the Malaysian water supply on the prevalence of fluorosis and dental caries. Community Dent Oral Epidemiol. 2018;46(5):492-9. 10.1111/cdoe.12407 30019792

[26] Zhang Y, Wang Y, Zhang Z, Wang Y, Jia J. Study on machine learning of molar incisor hypomineralization in an endemic fluorosis region in central China. Front Physiol. 2023;15(14):1088703. 10.3389/fphys.2023.1088703 PMC10050366 37008000

[27] Chouchene F, Masmoudi F, Baaziz A, Maatouk F, Ghedira H. Clinical status and assessment of caries on first permanent molars in a group of 6- to 13-year-old Tunisian school children. Clin Exp Dent Res. 2023;9(1):240-8. 10.1002/cre2.676 PMC9932250 36264010

[28] Weerheijm KL, Duggal M, Mejàre I, Papagiannoulis L, Koch G, Martens LC, et al. Judgement criteria for molar incisor hypomineralization (MIH) in epidemiologic studies: a summary of European meeting on MIH held in Athens, 2003. Eur J Paediatr Dent. 2003;3:110-3. 14529329

[29] Chagas FO, Valadas LAR, Dayo A, Dantas TCFB, Sorazabal A, Squassi A. Fluoride in drinking groundwater and prevalence of fluorosis in children and adolescents: a systematic review. Acta Odontol Latinoam. 2023;36(3):169-76. 10.54589/aol.36/3/169 PMC10867852 38345279

[30] Hung M, Hon ES, Mohajeri A, Moparthi H, Vu T, Jeon J, et al. A national study exploring the association between fluoride levels and dental fluorosis. JAMA Netw Open. 2023;6(6):e2318406. 10.1001/jamanetworkopen.2023.18406 PMC10290240 37351888

[31] Iheozor-Ejiofor Z, Walsh T, Lewis SR, Riley P, Boyers D, Clarkson JE, et al. Water fluoridation for the prevention of dental caries. Cochrane Database Syst Rev. 2024;10(10):CD010856. 10.1002/14651858.CD010856.pub3 PMC11449566 39362658

[32] Pérez-Pérez N, Irigoyen-Camacho ME, Boges-Yañez AS. Factors affecting dental fluorosis in low socioeconomic status children in Mexico. Community Dent Health. 2017;34(2):66-71. 10.1922/CDH_3981Perez-Perez06 28573833

[33] Aguilar-Díaz FC, Morales-Corona F, Cintra-Viveiro AC, de la Fuente-Hernández J. Prevalence of dental fluorosis in Mexico 2005-2015: a literature review. Salud Publica Mex. 2017;59(3):306-13. 10.21149/7764 28902318

