# Supplementary material for: Dental fluorosis and its association with molar incisor hypomineralization in children attending public schools: a cross-sectional study, São João da Boa Vista, 2023
Source: Epidemiol Serv Saude. 2026 Jun 19;35:e20251105. doi: 10.1590/S2237-96222026v35e20251105.en (PMC13686311; doi:10.1590/S2237-96222026v35e20251105.en)
Supplement: Translation

# Translation: Fluorose dentária e sua associação à hipomineralização molar-incisivo em escolares da rede pública: estudo transversal, São João da Boa Vista, 2023

**DOI:** 10.1590/S2237-96222026v35e20251105.pt

**Authors:** Giorgia dos Reis Doval, Sthefany Merlo Valverde Guezin, Letícia Mazeto Previero, Caio Luiz Lins-Candeiro, Gerson Aparecido Foratori-Junior, Luiz Renato Paranhos, Cristiane Maria da Costa Silva

**Affiliations:** 1 Centro Universitário das Faculdades Associadas de Ensino Faculdade de Odontologia São João da Boa Vista SP Brasil Centro Universitário das Faculdades Associadas de Ensino, Faculdade de Odontologia, São João da Boa Vista, SP, Brasil; 2 Universidade de São Paulo Faculdade de Odontologia de Bauru Bauru SP Brasil Universidade de São Paulo, Faculdade de Odontologia de Bauru, Bauru, SP, Brasil; 3 Universidade Federal de Uberlândia Faculdade de Odontologia Uberlândia MG Brasil Universidade Federal de Uberlândia, Faculdade de Odontologia, Uberlândia, MG, Brasil

**Keywords:** Prevalência, Fluorose Dentária, Inquérito Epidemiológico, Hipomineralização Incisivo-Molar, Estudo Transversal

## Abstract

**Objetivo:**

Avaliar a prevalência e a severidade da fluorose dentária em escolares e sua associação à hipomineralização molar-incisivo, à cárie e aos fatores socioeconômicos.

**Métodos:**

Estudo transversal, realizado com amostra probabilística de crianças de 7 a 11 anos matriculadas em escolas públicas de São João da Boa Vista, São Paulo. Consideraram-se três dimensões: exposição ambiental; características individuais e familiares; e desfechos clínicos. A prevalência e a severidade da fluorose foram avaliadas pelo índice de Dean, e a hipomineralização, pelos critérios da Academia Europeia de Odontopediatria. A cárie foi avaliada pelo índice de dentes cariados, perdidos e obturados. Dois examinadores calibrados realizaram os exames. Informações socioeconômicas foram obtidas dos responsáveis. Os dados foram analisados por meio de estatística descritiva e por regressão logística simples e múltipla, com significância de 5%.

**Resultados:**

Participaram deste estudo 473 crianças. A prevalência de fluorose foi de 28,5%. Predominaram as formas muito leves (11,0%) e leves (7,4%) às moderadas (3,4%) e severas (0,8%). A prevalência de hipomineralização foi de 12,7%, e 3,8% das crianças apresentavam ambos os defeitos de esmalte. A fluorose foi mais prevalente em escolares acima de 9 anos (*odds ratio*, OR 2,22; intervalo de confiança de 95%, IC95% 1,34; 3,66); em crianças sem hipomineralização molar-incisivo (OR 3,22; IC95% 1,20; 8,64); e entre aquelas cuja mãe estudou até o ensino médio (OR 2,15; IC95% 1,09; 4,23). Não houve associação com sexo, experiência de cárie e demais fatores socioeconômicos.

**Conclusão:**

A fluorose ocorreu predominantemente em formas leves, associando-se às características da criança e à escolaridade materna.

**Aspectos éticos**

Esta pesquisa respeitou os princípios éticos, obtendo os seguintes dados de aprovação:

Comitê de ética em pesquisa Centro Universitário das Faculdades Associadas de Ensino

Número do parecer 6.140.628

Data de aprovação 25/6/2023

Certificado de apresentação de apreciação ética 70686723.3.0000.5382

Registro do consentimento livre e esclarecido Obtido de todos os participantes antes da coleta.

## Introdução

A fluorose dentária é um dos principais efeitos adversos decorrentes da ingestão crônica de fluoreto durante o período da formação do esmalte dentário [1]. Essa condição impõe à saúde pública o desafio de equilibrar os benefícios do uso do fluoreto no controle da cárie dentária com a necessidade de minimizar os riscos associados à exposição excessiva [2,3]. Esse dilema é particularmente relevante em crianças em idade pré-escolar, que apresentam maior suscetibilidade à fluorose dentária por estarem em fase de mineralização do esmalte dentário e por terem maior risco de ingestão inadvertida de produtos fluoretados, como os dentifrícios [4-6].

A gravidade da fluorose depende tanto da quantidade quanto da duração da exposição ao fluoreto, podendo manifestar-se desde pequenas alterações na translucidez do esmalte até defeitos estruturais com impacto estético e funcional significativo [1,7-9]. Para além das manifestações clínicas, a condição pode comprometer a percepção de saúde bucal e afetar negativamente a autoestima de crianças e adolescentes, configurando um desafio que ultrapassa a esfera biológica e envolve as dimensões subjetivas do bem-estar [9,10].

Mesmo com o risco de fluorose, o fluoreto ainda é fundamental na prevenção da cárie, por atuar no equilíbrio entre desmineralização e remineralização do esmalte [6]. Essa eficácia justifica sua ampla utilização em diferentes veículos, como dentifrícios, soluções, géis e vernizes, e na fluoretação das águas de abastecimento público [6,8]. O desafio, portanto, não reside em uma fonte isolada, mas no controle da exposição cumulativa ao fluoreto proveniente de diversos veículos, incluindo produtos de higiene bucal e a fluoretação da água [11].

Nesse contexto, a vigilância epidemiológica é fundamental; ao monitorar a prevalência e a gravidade da fluorose dentária, pode-se obter informações sobre o nível de exposição populacional ao fluoreto. Assim, é possível identificar situações de ingestão excessiva e orientar ajustes nas políticas de fluoretação e em outras estratégias preventivas, de modo a garantir o equilíbrio entre benefícios e riscos [11,12]. 

No Brasil, em 2010, registrou-se a prevalência de 17% da fluorose dentária, chegando a 19% no Sudeste [13]. Esse dado é particularmente relevante pois essa região concentra maior número de municípios com abastecimento de água fluoretada e, ao mesmo tempo, melhores indicadores de saúde bucal em relação à cárie [14]. Além disso, chama a atenção a alta prevalência de hipomineralização molar-incisivo, defeito qualitativo do esmalte, de origem multifatorial. Assim como a fluorose, ela se manifesta clinicamente por alterações na translucidez e perdas estruturais, tornando os dentes mais suscetíveis à desmineralização e ao desenvolvimento acelerado da cárie [15]. 

Essas duas condições podem coexistir, embora tenham etiologias distintas, o que pode gerar vieses nas estimativas epidemiológicas [15,16]. A ocorrência de sobreposição clínica entre defeitos de esmalte em populações brasileiras [17,18] reforça a necessidade de critérios diagnósticos precisos para evitar classificações equivocadas em inquéritos epidemiológicos.

A experiência de cárie reflete padrões de exposição ao fluoreto, que influenciam o equilíbrio entre desmineralização e remineralização do esmalte dentário [6]. De modo semelhante, características sociodemográficas, como idade, sexo, renda familiar e escolaridade dos pais, podem moldar comportamentos em saúde, incluindo o uso e o acesso a produtos fluoretados, o que contribui para que haja variações nessa exposição [19,20]. Porém, no inquérito nacional mais recente, realizado em 2023, a fluorose dentária não foi monitorada [21]. Tal fato reforça a importância de estudos regionais para compreender o panorama atual da condição e subsidiar políticas públicas que assegurem tanto o controle da cárie dentária quanto a prevenção da fluorose dentária. 

Este estudo teve como objetivo avaliar a prevalência e a severidade da fluorose dentária em escolares da rede pública de São João da Boa Vista, São Paulo, bem como a sua associação à hipomineralização molar-incisivo, à cárie e aos fatores socioeconômicos.

## Métodos

### Desenho do estudo

Trata-se de estudo transversal elaborado de acordo com as recomendações da Organização Mundial de Saúde (OMS) [22]. O estudo foi estruturado a partir da organização das variáveis em três dimensões [23]: exposição ambiental ao fluoreto, representada pelo tipo de abastecimento de água no domicílio; características das crianças e dos familiares, incluindo renda familiar e escolaridade dos pais; e desfechos clínicos, compostos de hipomineralização molar-incisivo, experiência de cárie e fluorose, sendo esta última considerada a variável desfecho.

### Contexto

A pesquisa foi realizada em escolas públicas da zona urbana de São João da Boa Vista, São Paulo, entre outubro e dezembro de 2023. O município localiza-se a aproximadamente 220 km da capital paulista. Em 2022, a população era de 92.547 habitantes, e o município apresentava índice de desenvolvimento humano municipal de 0,79 e índice de Gini de 0,51. Em relação à infraestrutura, 92,2% dos domicílios dispunham de saneamento básico adequado [24], e o abastecimento público de água era universalizado e fluoretado. 

### Participantes

A amostra foi composta de escolares do ensino fundamental I, da rede pública da zona urbana de São João da Boa Vista, São Paulo, com idades entre 7 e 11 anos.

O pedido de autorização para realizar os exames com as crianças foi enviado aos pais ou responsáveis. Foram incluídos na amostra os estudantes cujos pais ou responsáveis autorizaram a participação, desde que apresentassem os quatro primeiros molares permanentes irrompidos, característica percebida no momento do exame epidemiológico. Os molares foram considerados irrompidos quando a face oclusal se apresentava exposta, sem recobrimento gengival. 

Foram excluídas crianças com síndromes ou condições que afetam a formação dentária (por exemplo, síndrome de Down, displasia ectodérmica e amelogênese imperfeita); com restaurações extensas ou desgastes severos que impedissem a avaliação; com manchas intrínsecas por tetraciclina ou manchas extrínsecas extensas não removíveis; em uso de aparelhos ortodônticos fixos; ou ausentes no dia do exame.

### Tamanho e seleção da amostra

O cálculo amostral considerou nível de confiança de 95%, prevalência estimada de fluorose em 50%, erro de 5% e efeito de delineamento de 1,^2^, devido à amostragem por conglomerados. A amostra mínima estimada foi de 415 estudantes, acrescida de 20% para compensar recusas, totalizando 498 participantes previstos. 

A seleção das escolas foi realizada por sorteio aleatório. O município foi dividido em cinco regiões administrativas (exceto o distrito industrial), sendo todas as escolas identificadas e sorteadas de forma proporcional ao número de alunos matriculados em cada área. A região central, por ser a mais populosa, foi representada por mais de uma instituição. Ao final, seis escolas foram selecionadas, assegurando a representatividade da zona urbana do município.

### Variáveis

Para o desfecho principal, as varáveis foram presença e severidade da fluorose dentária (índice de Dean) [19]. Os dentes foram classificados em seis categorias, de acordo com o índice de Dean [22], a saber:

- normal (0) – ausência de alterações;
- questionável (1) – esmalte mostra pequena diferença com relação à translucidez normal do esmalte e que pode variar de poucas manchas esbranquiçadas a pontos ocasionais;
- muito leve (2) – pequenas áreas opacas e esbranquiçadas, envolvendo menos de 25% da superfície dental;
- leve (3) – opacidades mais extensas, porém afetando menos de 50% da superfície;
- moderada (4) – esmalte com manchas difusas e desgaste, podendo incluir pigmentação amarronzada; e
- severa (5) – hipomineralização marcante, com alteração de forma do dente, manchas extensas e, por vezes, defeitos estruturais.

A fluorose dentária foi definida como a presença de qualquer grau de alteração do esmalte, sendo considerados como casos os indivíduos com escore do índice de Dean>0 [25]. A inclusão dos graus mais leves de fluorose foi metodologicamente consistente com estudos prévios que investigaram a associação entre fluorose dentária e hipomineralização molar-incisivo [16,26] e outros defeitos de esmalte [27]. Isso contribuiu para uma avaliação mais precisa das inter-relações entre defeitos do esmalte.

As variáveis clínicas independentes foram experiência de cárie de acordo com a OMS por meio do índice de dentes cariados, perdidos e obturados [22] e hipomineralização molar-incisivo, avaliada segundo critérios da Academia Europeia de Odontopediatria [28]. Foram considerados casos de hipomineralização molar-incisivo quando se evidenciou ao menos um primeiro molar permanente com opacidades demarcadas (brancas, amarelas ou acastanhadas) maiores de 1 mm; fratura pós-eruptiva; restaurações atípicas; e exodontia atribuída à hipomineralização molar-incisivo. Essas alterações precisavam estar presentes em pelo menos um primeiro molar permanente, podendo ou não envolver incisivos.

As variáveis sociodemográficas foram sexo, idade, renda familiar, escolaridade dos pais e acesso à água tratada.

### Fontes de dados e procedimentos de coleta

Os exames clínicos foram realizados nas escolas seguindo as recomendações operacionais da OMS para inquéritos epidemiológicos [22]. Os exames foram realizados sob iluminação natural, com criança e examinador sentados. Utilizaram-se espelho plano nº 5, espátulas de madeira e sonda periodontal com ponta esférica (sonda OMS), utilizada apenas para remover resíduos visíveis. Previamente, as crianças realizaram escovação supervisionada. Todos os dentes presentes na cavidade bucal foram avaliados, tanto decíduos quanto permanentes. Entretanto, a classificação da hipomineralização molar-incisivo foi realizada exclusivamente nos dentes permanentes. Dois examinadores treinados e calibrados conduziram os exames. Os dados foram registrados em fichas específicas. 

As variáveis sociodemográficas foram obtidas por meio de questionário estruturado, enviado e respondido pelos pais ou responsáveis. O instrumento contemplou informações básicas das crianças (sexo e idade) e dados de caracterização familiar, como renda familiar mensal, categorizada de acordo com faixas de salários mínimos, escolaridade dos pais, classificada em níveis formais de ensino e tipo de abastecimento de água no domicílio (rede geral de distribuição/água tratada, poço/nascente, outros). 

### Esforços para minimizar vieses

Para reduzir vieses diagnósticos, em casos de dúvida quanto à presença de fluorose, os dentes foram classificados como normais. O mesmo procedimento foi adotado para cárie e hipomineralização molar-incisivo, a fim de evitar superestimação das prevalências. Os examinadores passaram por processo de treinamento e calibração, composto de etapas teóricas e práticas. 

Inicialmente, foi realizada uma fase teórica, com estudo prévio dos índices utilizados, análise de imagens clínicas projetadas em slides e discussão de situações que poderiam ser encontradas durante o trabalho de campo. Essa etapa visou padronizar os critérios diagnósticos de fluorose, hipomineralização molar-incisivo e cárie dentária entre os examinadores. Posteriormente, ocorreu o treinamento prático utilizando um banco de imagens intrabucais padronizadas, representando diferentes condições clínicas de fluorose, cárie e hipomineralização. 

Os examinadores registraram seus diagnósticos nas mesmas fichas clínicas padronizadas empregadas na pesquisa, e as divergências identificadas foram discutidas até se alcançar consenso. O treinamento totalizou oito horas. Após essa etapa, foi realizada a calibração clínica. Para isso, os examinadores avaliaram 25 crianças em dentição mista, selecionadas por conveniência de uma escola não sorteada para compor a amostra final. 

Após o intervalo de uma semana, as mesmas crianças foram reavaliadas para estimar a concordância inter e intraexaminadores. A reprodutibilidade diagnóstica foi mensurada pelo coeficiente Kappa, que indicou alta concordância interexaminadores para cárie (Kappa 0,81), fluorose (Kappa 0,89) e hipomineralização molar-incisivo (Kappa 0,91). Os valores de Kappa intraexaminadores foram superiores a 0,81.

### Análise de dados

Foram realizadas análises descritivas por meio de frequências absolutas e relativas. Depois, foram ajustados modelos de regressão logística simples e múltiplo para analisar as associações com o desfecho presença de fluorose dentária. A qualidade do modelo foi analisada pelo Critério de Informação de Akaike. Foram então estimadas as razões de chances (*odds ratio*, OR) brutas e ajustadas, com os respectivos intervalos de confiança (IC95%). Todas as análises foram realizadas no programa R (R Core Team, 2025).

## Resultados

Foram sorteadas 6 escolas públicas da zona urbana de São João da Boa Vista, São Paulo, garantindo a representatividade das diferentes regiões administrativas do município, com exceção do distrito industrial. No total, 738 crianças de 7 a 11 anos estavam matriculadas nessas instituições e, portanto, eram elegíveis para o estudo. Destas, 498 foram convidadas aleatoriamente a participar, por meio de sorteio simples. O número de participantes por escola foi proporcional ao total de matriculados. A amostra final foi composta de 473 escolares, todos com dados completos para fluorose, hipomineralização molar-incisivo e cárie (Figura 1). Resultou-se em uma taxa de resposta de 94,9%.

**Figura 1:** Seleção da amostra do estudo. São João da Boa Vista, 2023 (n=473)

A idade média dos participantes foi 8,9±1,2 anos, variando entre 7 e 11 anos (mediana 9 anos), sendo 52,6% do sexo masculino. A maioria das famílias possuía renda entre 1 e 3 salários mínimos (53,5%), e grande parte das mães tinha ensino médio completo (48,7%). Em relação ao abastecimento domiciliar, 86,0% relataram utilizar água da rede geral tratada pela Companhia de Saneamento Básico de Estado de São Paulo, enquanto 6,8% consumiam água de poço ou nascente (Tabela 1).

**Tabela 1:** Análise descritiva das variáveis de perfil sociodemográfico da amostra em frequências absoluta (n) e relativa (%). São João da Boa Vista, 2023 (n=473)

Variáveis	Frequências n (%)
Sexo	
Feminino	224 (47,4)
Masculino	249 (52,6)
**Idade **(anos)	
7	68 (14,4)
8	120 (25,4)
9	110 (23,3)
10	111 (23,5)
11	64 (13,5)
**Renda familiar mensal **(**salários mínimos**)	
Sem renda	21 (4,4)
Até 1	113 (23,9)
De 1-2	156 (33,0)
Mais de 2-3	97 (20,5)
Mais de 3-5	39 (8,2)
Mais de 5-10	8 (1,7)
Mais de 10-20	1 (0,2)
Mais de 20	1 (0,2)
Sem informação	37 (7,8)
**Grau de instrução do pai**	
Não alfabetizado	5 (1,1)
Alfabetizado	32 (6,8)
Até ensino fundamental I completo	26 (5,5)
Até o ensino fundamental II completo	47 (9,9)
Até o ensino médio completo	86 (18,2)
Superior incompleto	142 (30,0)
Superior completo	20 (4,2)
Sem informação	115 (24,3)
**Grau de instrução da mãe**	
Não alfabetizada	4 (0,8)
Alfabetizada	4 (0,8)
Até ensino fundamental I completo	17 (3,6)
Até ensino fundamental II completo	82 (17,4)
Até o ensino médio completo	230 (48,7)
Superior incompleto	45 (9,5)
Superior completo	54 (11,4)
Sem informação	37 (7,8)
**Água utilizada na casa**	
Rede geral de distribuição (água tratada)	407 (86,0)
Poço ou nascente (mina de água)	32 (6,8)
Outros	3 (0,6)
Sem informação	31 (6,6)

A prevalência de fluorose dentária foi de 28,5%, com predomínio das formas muito leves (11,0%). Casos moderados (3,4%) e severos (0,8%) também foram observados. A prevalência de hipomineralização molar-incisivo foi de 12,7%, e 3,8% da amostra total (13,3% entre os casos de fluorose) apresentaram ambas as condições simultaneamente. (Tabela 2). Considerando conjuntamente as dentições decídua e permanente, 50,5% das crianças apresentaram experiência de cárie (Tabela 3).

**Tabela 3:** Frequências absoluta (n) e relativa (%), razões de chance (*odds ratio*, OR) bruta e ajustada e intervalos de confiança de 95% (IC95%) para a presença de fluorose dentária segundo variáveis clínicas e sociodemográficas. São João da Boa Vista, 2023 (n=473)

Variáveis	Total n (%)	Presença de fluorose n (%)	OR bruta (IC95%)	p-valor	OR ajustada (IC95%)	p-valor
Global	473 (100,0)	135 (28,5)				
Sexo						
Feminino	224 (47,4)	66 (29,5)	1,09 (0,73; 1,62)	0,673	1,25 (0,76; 2,04)	0,379
Masculino	249 (52,6)	69 (27,7)	Referência		Referência	
**Faixa etária **(anos)						
Até 9	298 (63,0)	65 (21,8)	Referência		Referência	
>9	175 (37,0)	70 (40,0)	2,39 (1,59; 3,60)	<0,001	2,22 (1,34; 3,66)	0,002
**Renda familiar **(**salários mínimos**)						
Até 2	290 (61,3)	82 (28,3)	Referência		Referência	
Mais de 2	146 (30,9)	44 (30,1)	1,09 (0,71; 1,69)	0,686	1,21 (0,71; 2,07)	0,485
Sem informação	37 (7,8)	9 (24,3)				
**Grau de instrução do pai**						
Até 2º grau completo	306 (64,7)	83 (27,1)	0,84 (0,44; 1,59)	0,587	0,58 (0,27; 1,25)	0,164
Superior	52 (11,0)	16 (30,8)	Referência		Referência	
Sem informação	115 (24,3)	36 (31,3)				
**Grau de instrução da mãe**						
Até 2º grau completo	337 (71,2)	101 (30,0)	1,34 (0,80; 2,24)	0,269	2,15 (1,09; 4,23)	0,026
Superior	99 (20,9)	24 (24,2)	Referência		Referência	
Sem informação	37 (7,8)	10 (27,0)				
**Acesso à água tratada**						
Rede geral de distribuição (água tratada)	407 (92,1)	120 (29,5)	1,81 (0,73; 4,51)	0,202	1,50 (0,57; 3,98)	0,413
Poço ou nascente (mina de água)	32 (6,8)	6 (18,8)	Referência		Referência	
Outros	3 (0,6)	0 (0,0)				
Sem informação	31 (6,6)	9 (29,0)				
**Experiência de cárie**						
Ceod^1^ e CPOD^2^=0	234 (49,5)	78 (33,3)	1,60 (1,07; 2,39)	0,023	1,38 (0,84; 2,28)	0,208
Ceod ou CPOD>0	239 (50,5)	57 (23,8)	Referência		Referência	
**Presença de hipomineralização molar**-incisivo						
Não	413 (87,3)	127 (30,8)	2,89 (1,33; 6,25)	0,007	3,22 (1,20; 8,64)	0,020
Sim	60 (12,7)	8 (13,3)	Referência		Referência	

^1^Ceod: dentes decíduos cariados, extraídos e obturados; ^2^CPOD: dentes permanentes cariados, perdidos e obturados. A qualidade do modelo foi analisada pelo Critério de Informação de Akaike (AIC), sendo AIC(modelo vazio)=411,23 e AIC(modelo final)=397,82.

**Tabela 2:** Análise descritiva das variáveis clínicas: severidade de fluorose dentária, hipomineralização molar-incisivo e experiência de cárie na dentição decídua e permanente, apresentadas em frequências absoluta (n) e relativa (%). São João da Boa Vista, 2023 (n=473)

Variáveis	Frequências n (%)
**Severidade de fluorose**	
Normal	338 (71,5)
Questionável	35 (7,4)
Muito leve	52 (11,0)
Leve	28 (5,9)
Moderada	16 (3,4)
Severa	4 (0,8)
**Hipomineralização molar-incisivo **(HMI)	
HMI=0	413 (87,3)
HMI>0	60 (12,7)
**Experiência de cárie na dentição decídua**	
Ceod^1^=0	277 (58,6)
Ceod>0	197 (41,4)
**Experiência de cárie na dentição permanente **	
CPOD=0	389 (82,2)
CPOD>0	84 (17,8)

^1^Ceod: dentes decíduos cariados, extraídos e obturados ; ^2^CPOD: dentes permanentes cariados, perdidos e obturados.

Na análise de regressão logística múltipla, a fluorose foi significativamente mais frequente em escolares:

- com idade maior que 9 anos (OR 2,22; IC95% 1,34; 3,66);
- sem hipomineralização molar-incisivo (OR 3,22; IC95% 1,20; 8,64); e
- entre aqueles cujas mães estudaram até o ensino médio completo (OR 2,15; IC95% 1,09; 4,23).

Embora a prevalência de fluorose tenha sido maior entre crianças que consumiam água da rede geral (29,5%) em comparação às que utilizavam poço ou nascente (18,8%), a diferença não foi significativa (p-valor 0,201). Não houve associação significativa com sexo, fonte de água utilizada ou experiência de cárie após o ajuste (Tabela 3).

## Discussão

A prevalência de fluorose dentária encontrada neste estudo foi de 28%, com predomínio das formas leves. Observou-se que as variáveis das dimensões “características individuais” e “desfechos clínicos” apresentaram associação significativa com a condição. Crianças acima de 9 anos apresentaram maior chance de fluorose, o que se justifica pelo maior número de dentes permanentes irrompidos e, portanto, maior visibilidade clínica dos defeitos do esmalte. Na dimensão dos desfechos clínicos, identificou-se associação inversa entre fluorose e hipomineralização molar-incisivo. Não foram observadas associações com sexo, experiência de cárie, renda familiar ou tipo de abastecimento de água.

Algumas limitações deste estudo devem ser consideradas na interpretação dos resultados. O delineamento transversal não permite estabelecer relações de causalidade, o que restringe as conclusões em relação às associações observadas. Utilizar questionários respondidos pelos pais ou responsáveis pode ter introduzido vieses de informação, decorrentes da conscientização gerada pelo próprio estudo. Os exames clínicos, realizados em ambiente escolar, também estão sujeitos a limitações relacionadas à iluminação e à secagem dos dentes, o que pode influenciar a acurácia diagnóstica. A amostra contemplou apenas escolares da rede pública urbana, o que reduz a possibilidade de generalização para outros contextos sociais e geográficos. 

No entanto, o rigor metodológico adotado, incluindo o processo de calibração com elevado valor de Kappa, critérios diagnósticos padronizados e amostragem probabilística, contribui para a solidez dos resultados encontrados. Este estudo reforçou a necessidade de monitoramento contínuo da fluorose, especialmente em suas formas moderada e severa, dado seu potencial impacto estético e psicossocial [8,9].

Neste estudo, 4% das crianças com fluorose apresentaram simultaneamente hipomineralização molar-incisivo. Do ponto de vista etiológico, são condições distintas: a fluorose é decorrente da exposição excessiva ao fluoreto durante a formação do esmalte, enquanto a hipomineralização molar-incisivo resulta da interação entre fatores sistêmicos e predisposição genética que podem interferir na atividade ameloblástica durante a fase de maturação dentária [15]. Embora ambas sejam defeitos qualitativos do esmalte, elas apresentam manifestações clínicas distintas. A fluorose se manifesta por opacidades difusas, de distribuição simétrica pela dentição, enquanto a hipomineralização molar-incisivo se caracteriza por lesões bem delimitadas no esmalte, com distribuição assimétrica. 

A manifestação clínica de ambas as condições depende da severidade e podem variar desde opacidades brancas no esmalte até perdas estruturais, em casos mais severos [7,15,28], o que torna o diagnóstico diferencial desafiador quando ambos os defeitos estão presentes. Essa é uma preocupação particularmente relevante em estudos de campo, nos quais a sobreposição dos defeitos pode levar à superestimação ou à subestimação da prevalência [15-18]. No México, em 2023, tal preocupação foi evidenciada ao se demonstrar a relação inversa entre fluorose e hipomineralização molar-incisivo em escolares expostos a altas concentrações de flúor na água. Sugerem-se mecanismos etiológicos distintos, mas que podem se sobrepor clinicamente [16].

Neste estudo, o elevado índice Kappa (≥0,81) demonstrou excelente confiabilidade diagnóstica, reforçando a robustez das avaliações clínicas realizadas. No entanto, a falta de critérios rigorosos pode comprometer tanto os inquéritos epidemiológicos quanto a definição de estratégias preventivas e terapêuticas [15]. Assim, a coexistência observada neste estudo reforçou a necessidade de protocolos diagnósticos padronizados e treinamento adequado dos examinadores para garantir estimativas confiáveis e subsidiar decisões em saúde bucal coletiva.

Nesta análise, a variável “fonte de água utilizada na residência” não apresentou associação estatisticamente significativa com a ocorrência de fluorose (p-valor 0,413). No entanto, no Brasil, em 2019, a fonte de água foi identificada como um determinante importante, sobretudo em áreas abastecidas por poços artesianos. Nesse caso, as prevalências de fluorose chegaram a 52% [7]. Em 2023, em países da África e da Ásia, elas ultrapassaram 90% [29]. Por outro lado, mesmo em contextos urbanos com ampla cobertura de água tratada, concentrações próximas do ideal estão associadas à maior prevalência da condição, sendo esperado que 40% da população apresente algum grau de fluorose [30,31]. A homogeneidade da amostra nesta análise, com 86% consumindo água fluoretada, provavelmente explica a ausência de associação encontrada, embora não exclua a importância do monitoramento contínuo da qualidade da água [14,19].

O predomínio de casos leves encontrado é consistente com o padrão descrito para populações expostas à água fluoretada dentro dos níveis recomendados [30]. Entretanto, a ocorrência de formas moderadas e severas chama a atenção para possíveis fragilidades no controle operacional da fluoretação e para o papel das exposições cumulativas durante a infância [1,32] amplamente discutidas na literatura, mas ainda pouco monitoradas no Brasil.

A relação entre fluorose e condições socioeconômicas é multifacetada e dependente do contexto. No México, em 2014, registrou-se elevada prevalência de fluorose, inclusive em áreas com concentrações de flúor na água dentro do nível considerado ótimo. Isso reforça a contribuição de outras fontes de exposição, como o uso precoce de dentifrícios fluoretados e o consumo de bebidas industrializadas [32], práticas mais comuns em populações com melhores condições socioeconômicas. Em contrapartida, a instabilidade da fluoretação do abastecimento e a dependência de água subterrânea estão associadas a contextos de menor desenvolvimento socioeconômico, refletindo desigualdades subjacentes que amplificam a carga da condição [19,20,29]. 

Em 2017, também no México, identificou-se que a ocorrência de formas severas de fluorose em regiões mais vulneráveis foi mais frequente em crianças cujas mães tinham menor escolaridade (OR 1,71; p-valor 0,007) [32], o que pode ser resultado de exposições não controladas e capacidade limitada de mitigação [33]. Nesse cenário heterogêneo, a escolaridade materna emerge como um marcador social relevante da exposição ao fluoreto. Mães com menor escolaridade podem apresentar menor acesso à informação e aos recursos que permitam o manejo adequado da exposição infantil ao fluoreto. 

Mesmo quando a concentração de flúor na água de abastecimento é semelhante, diferenças comportamentais associadas à escolaridade materna podem resultar em padrões distintos de exposição cumulativa ao fluoreto ao longo da infância [33]. Esse resultado tensiona a noção de que políticas universais, como a fluoretação das águas de abastecimento, são suficientes para garantir proteção equitativa evidenciando a necessidade de estratégias complementares voltadas a grupos socialmente mais vulneráveis, mesmo em contextos de ampla cobertura de água fluoretada.

A prevalência de fluorose dentária em crianças brasileiras de 12 anos, em 2010, foi de 17%, predominando as formas muito leves (11%) e leves (4%) [14]. A fluorose moderada representou menos de 2% da amostra nacional, enquanto a forma severa apresentou ocorrência residual [13]. No Sudeste, em 2010, a prevalência de fluorose foi mais elevada (19,1%). Neste estudo, realizado em São João da Boa Vista, em 2023, identificou-se prevalência de 28%, com proporção mais expressiva de casos moderados e severos do que a descrita tanto para o país quanto para a região em 2010. Esses achados sugerem que a dimensão ambiental e a falta de monitoramento sistemático da fluorose podem contribuir para mudanças no perfil epidemiológico dessa condição ao longo do tempo. O fato de não haver investigação da fluorose no último inquérito epidemiológico nacional, realizado em 2023, [21] dificulta comparações e limita a compreensão da tendência temporal dessa condição no país. 

Os resultados deste estudo não apenas estabelecem uma linha de base para o município, mas também levantam a hipótese de que o panorama da fluorose no país se transformou ao longo dos últimos anos, o que implica diretamente a saúde pública. Futuros estudos multicêntricos e a retomada do monitoramento em inquéritos nacionais são fundamentais para confirmar essa tendência e orientar políticas públicas de vigilância em saúde.

A prevalência de fluorose dentária encontrada neste estudo entre escolares da rede pública de ensino, com predominância de formas leves, foi superior às médias nacional e regional. Identificou-se associação inversa significativa entre fluorose e hipomineralização molar-incisivo. Observa-se que, embora apresentem etiologias distintas, ambas as condições podem coexistir.

A relação entre fluorose dentária e menor escolaridade materna evidencia o papel dos determinantes sociais na distribuição dessa condição. Esses achados reforçaram a importância da vigilância epidemiológica da fluorose dentária, em especial em contextos de ampla cobertura de água fluoretada e apontaram para a necessidade de estratégias preventivas que considerem o papel das desigualdades sociais exposição cumulativa ao fluoreto durante a infância. 

## Data Availability

Os dados utilizados na pesquisa estão disponíveis em: https://data.mendeley.com/datasets/23dvmb8yt4/1.
